# Differentiation between benign and malignant vertebral compression fractures using qualitative and quantitative analysis of a single fast spin echo T2-weighted Dixon sequence

**DOI:** 10.1007/s00330-021-07947-1

**Published:** 2021-05-26

**Authors:** Sebastien Bacher, Steven David Hajdu, Yael Maeder, Vincent Dunet, Tom Hilbert, Patrick Omoumi

**Affiliations:** 1 Department of Radiology, Lausanne University Hospital and University of Lausanne, Rue du Bugnon 46, 1011 Lausanne, Switzerland; 2 Advanced Clinical Imaging Technology, Siemens Healthcare AG, Lausanne, Switzerland; 3 LTS5 , École Polytechnique Fédérale de Lausanne (EPFL) , Lausanne, Switzerland

**Keywords:** Magnetic resonance imaging, Spinal fractures, Compression fractures, Bone marrow, Diagnostic imaging

## Abstract

**Objectives:**

To determine and compare the qualitative and quantitative diagnostic performance of a single sagittal fast spin echo (FSE) T2-weighted Dixon sequence in differentiating benign and malignant vertebral compression fractures (VCF), using multiple readers and different quantitative methods.

**Methods:**

From July 2014 to June 2020, 95 consecutive patients with spine MRI performed prior to cementoplasty for acute VCFs were retrospectively included. VCFs were categorized as benign (*n* = 63, mean age = 76 ± 12 years) or malignant (*n* = 32, mean age = 63 ± 12 years) with a best valuable comparator as a reference. Qualitative analysis was independently performed by four radiologists by categorizing each VCF as either benign or malignant using only the image sets provided by FSE T2-weighted Dixon sequences. Quantitative analysis was performed using two different regions of interest (ROI1-2) and three methods (signal drop, fat fraction (FF) from ROIs, FF maps). Diagnostic performance was compared using ROC curves analyses. Interobserver agreement was assessed using kappa statistics and intraclass correlation coefficients (ICC).

**Results:**

The qualitative diagnostic performance ranged from area under the curve (AUC) = 0.97 (95% CI: 0.91–1.00) to AUC = 0.99 (95% CI: 0.95–1.0). The quantitative diagnostic performance ranged from AUC = 0.82 (95% CI: 0.73–0.89) to AUC = 0.97 (95% CI: 0.91–0.99). Pairwise comparisons showed no statistical difference in diagnostic performance (all *p* > 0.0013, Bonferroni-corrected *p* < 0.0011). All five cases with disagreement among the readers were correctly diagnosed at quantitative analysis using ROI2. Interobserver agreement was excellent for both qualitative and quantitative analyses.

**Conclusions:**

A single FSE T2-weighted Dixon sequence can be used to differentiate benign and malignant VCF with high diagnostic performance using both qualitative and quantitative analyses, which can provide complementary information.

**Key Points:**

• *Qualitative analysis of a single FSE T2-weighted Dixon sequence yields high diagnostic performance and excellent observer agreement for differentiating benign and malignant compression fractures.*

• *The same FSE T2-weighted Dixon sequence allows quantitative assessment with high diagnostic performance.*

• *Quantitative data can readily be extracted from the FSE T2-weighted Dixon sequence and may provide complementary information to the qualitative analysis, which may be useful in doubtful cases.*

## Introduction

Magnetic resonance imaging (MRI) of the spine is widely used to detect and characterize vertebral compression fractures (VCFs), which remain highly prevalent in the population [1]. In order to initiate appropriate treatment and improve outcome, it is important for clinicians to differentiate between benign osteoporotic and malignant VCFs, which can be challenging in practice [2, 3]. In a recent meta-analysis, qualitative assessment of MRI was shown to be highly accurate in differentiating benign from malignant VCFs, with sensitivity and specificity reported at 89% and 88%, respectively [4].

A standard MRI protocol of the spine typically consists of fast spin echo (FSE) T1-, T2-weighted (T2w) and fat-suppressed fluid-sensitive sequences. Compared to other fat-suppressed fluid-sensitive sequences currently available, the advantages of the Dixon technique have already been highlighted in the literature and include more homogenous fat suppression in large field-of-view acquisitions than chemical shift selective (CHESS) methods, higher signal-to-noise ratio than short-tau inversion recovery (STIR), and multiple image sets with different contrasts generated from a single acquisition [5–10]. The image sets derived from a T2w Dixon acquisition include in-phase, out-of-phase, fat-only, and water-only. The derived fat-only images offer an additional benefit: they may replace T1-weighted sequences for the study of bone marrow fat in some indications [11, 12]. This has allowed the simplification of protocols for several applications, including the detection of bone marrow metastases, sacroiliitis, or for the workup of low back pain and/or lumbar radiculopathy [11–13]. However, the diagnostic performance of the morphological assessment of a simplified protocol using a single FSE T2w Dixon sequence in the differentiation between benign and malignant VCFs has not yet been assessed.

In order to further improve the diagnostic performance of MRI in characterizing VCFs, some authors have successfully used quantitative chemical shift water-fat imaging [14–16]. This method provides a measurement of the fat fraction, which is decreased in malignant VCFs due to the replacement of the normal fatty component of the bone marrow by tumoral tissue, unlike benign VCFs where fat is preserved. Quantitative water-fat imaging can be used with different types of pulse sequences and has been validated for the differentiation of benign and malignant VCFs using gradient echo sequences [2, 15, 17]. However, the use of these quantitative methods requires the acquisition of dedicated sequences in addition to the standard protocol. To the best of our knowledge, the performance of quantitative analysis based on *FSE* T2w Dixon sequences for the differentiation of benign and malignant VCFs has not yet been reported.

We hypothesized that a single FSE T2 Dixon sequence could provide high diagnostic performance for both morphological and quantitative characterization of VCFs. Therefore, the purpose of this study was (1) to determine the diagnostic performance of a single FSE T2w Dixon sequence in qualitatively differentiating benign and malignant VCFs using multiple readers with different backgrounds, (2) to determine the diagnostic performance of quantitative analysis of the same sequence in differentiating benign and pathological VCFs using different quantification methods, (3) to compare the diagnostic performance of qualitative and quantitative analyses, and (4) to assess the added value of quantitative analysis in discordant cases at qualitative analysis.

## Methods

### Study protocol

**Fig. 1:**
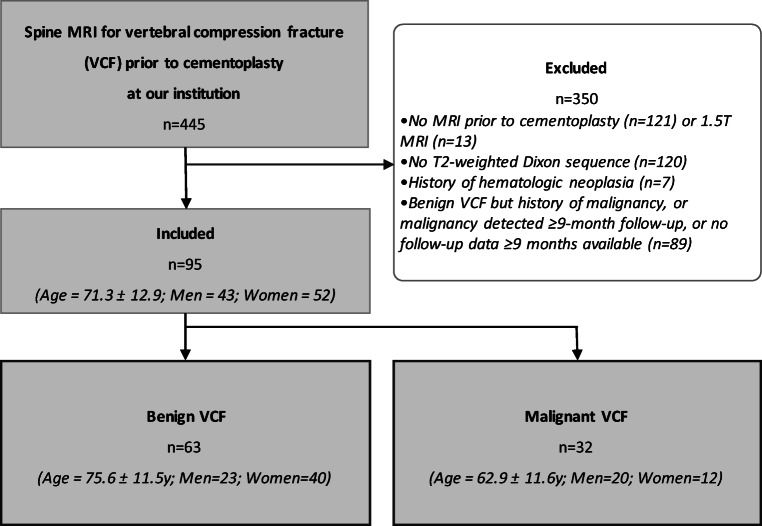
Flowchart shows patients selection criteria and group characteristics

The local institutional review board (Swiss Ethics Committees on research involving humans *#*2019-00879) approved this monocentric retrospective observational cohort study. Informed consent was waived for study participants who had not signed the general research agreement of our institution. From July 2014 to June 2020, we retrospectively included 445 consecutive adult patients treated for an acute vertebral compression fracture (VCF) following our institutional guidelines for percutaneous treatment of VCF, and who underwent 3.0T MRI in our department a maximum of four weeks prior to cementoplasty [18]. Figure 1 summarizes selection criteria and patients’ characteristics.

### MR imaging

All imaging was performed on 3-T scanners (MAGNETOM Skyra, Skyra^Fit^, Prisma^Fit^, Verio; Siemens Healthcare) without hardware adjustments and with standard radiofrequency body transmit coils and spine receive coils. A FSE T2w two-point Dixon was acquired for all patients in the sagittal plane. A conventional FSE sequence was used to acquire the in-phase contrasts. This acquisition was automatically repeated with the read-outs shifted in order to sample the k-space center 1.1 ms (0.5/440 Hz) before each spin echo, resulting in the out-of-phase image. Acquisition parameters were as follows: TR = 4600–5340 ms, TE = 77–94 ms and flip angle = 140°, bandwidth = 340 Hz/pixel, IPAT factor: 2. Twenty-one sagittal slices were reconstructed with 3 mm slice thickness and 0.3 mm gap. The field of view was 260 mm^2^ (voxel size of 0.81 × 0.81 × 3.0 mm^3^). Four sets of images were automatically reconstructed from the FSE T2w Dixon sequence: in-phase, out-of-phase, water-only, and fat-only. Additional sequences were used according to clinical need, but not used in this study.

### Reference standard to categorize VCFs as benign or malignant

VCFs were categorized as benign or malignant based on a best valuable comparator consisting of a consensus reading performed by three observers (Y.M., S.H., P.O.) after the end of readings of all available medical records, radiographs, CT, MRI, bone scans and PET-CT studies, and biopsy data (biopsy of target vertebra performed during cementoplasty).

For VCFs categorized as benign according to the best valuable comparator, a follow-up of nine months or more was required, in particular to avoid false negative results of biopsy. VCFs were therefore considered benign if fulfilling all of the following criteria: no current or past history of malignancy, no positive biopsy result (biopsy could be absent or negative), no malignancy found at clinical and imaging follow-up of nine months or more.

VCFs were considered malignant if the best valuable comparator based on all data available was suggestive of a malignant origin. No minimum follow-up period was required.

### Qualitative assessment

**Fig. 2:**
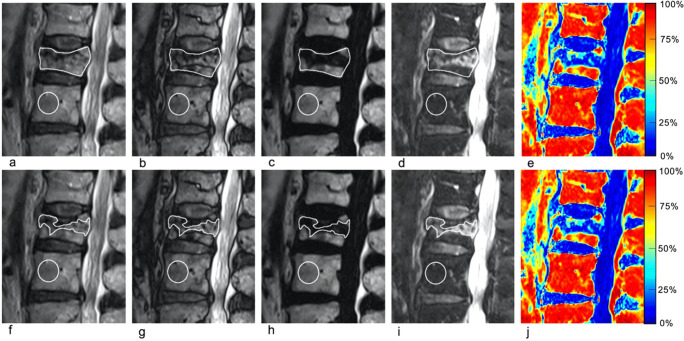
75-year-old woman with a benign vertebral compression fracture (VCF) (**a**–**j**). Sagittal thoracic spine MR images generated from a single FSE T2-weighted Dixon sequence include in-phase (**a** and **f**), out-of-phase (**b** and **g**), fat-only (**c** and **h**), water-only images (**d** and **i**), and fat fraction (FF) maps (**e** and **j**). Region of interest (ROI)1 (**a**–**e**) was drawn on the entire vertebra at the level of the VCF on (**a**) in-phase image and copy-pasted onto the (**b**) out-of-phase, (**c**) fat-only, (**d**) water-only images, and (**e**) fat fraction maps. ROI2 (**f**–**j**) was drawn on the area with high signal intensity on the (**i**) water-only image, and copy-pasted onto the (**f**) in-phase, (**g**) out-of-phase, (**h**) fat-only images, and (**j**) fat fraction maps. Control ROI (circle) placed on an adjacent healthy vertebra and is depicted on all images. In this benign VCF, signal drop, FF calculated from water-only and fat-only images, and FF from maps were ROI1: 50.4%/ROI2: 47.6%; ROI1: 51.4%/ROI2: 36.2%; and ROI1: 39.8%/ROI2: 31.3%, respectively

**Fig. 3:**
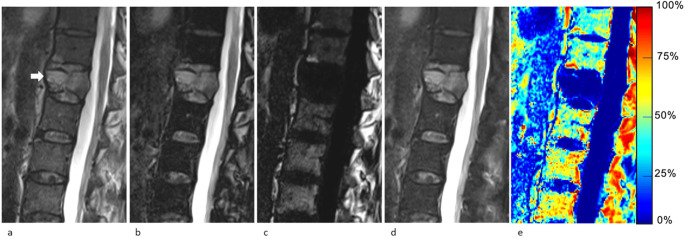
63-year-old man with a malignant vertebral compression fracture (VCF) at T12 level (arrow). Sagittal lumbar spine MR images generated from a signal FSE T2-weighted Dixon sequence include (**a**) in-phase, (**b**) out-of-phase, (**c**) fat-only, (**d**) water-only images, and (**e**) fat fraction (FF) map. The VCF was correctly characterized at qualitative assessment by all readers and at quantitative assessment by all measurement methods using the cutoffs determined in this study (signal drop, FF calculated from water-only and fat-only images, and FF from maps were ROI1: 0.8%/ROI2: 0.7%; ROI1: 0.8%/ROI2: 0.7%; and ROI1: 2.4%/ROI2: 1.0%, respectively)

**Fig. 4:**
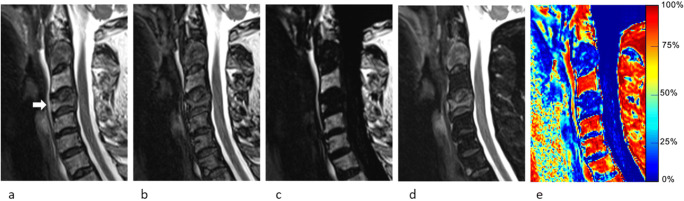
71-year-old man with a malignant vertebral compression fracture (VCF) at C4 level (arrow). Sagittal cervical spine MR images generated from a signal FSE T2-weighted Dixon sequence include (**a**) in-phase, (**b**) out-of-phase, (**c**) fat-only, (**d**) water-only images, and (**e**) fat fraction (FF) map. While readers all agreed on the malignant nature of the VCF, quantitative analysis was falsely negative for signal drop with both ROIs using the cutoffs determined in this study (signal drop, FF calculated from water-only and fat-only images, and FF from maps were ROI1: 25.8%/ROI2: 25.4%; ROI1: 14.3%/ROI2: 1.7%; and ROI1: 12.2%/ROI2: 10.9%, respectively)

**Fig. 5:**
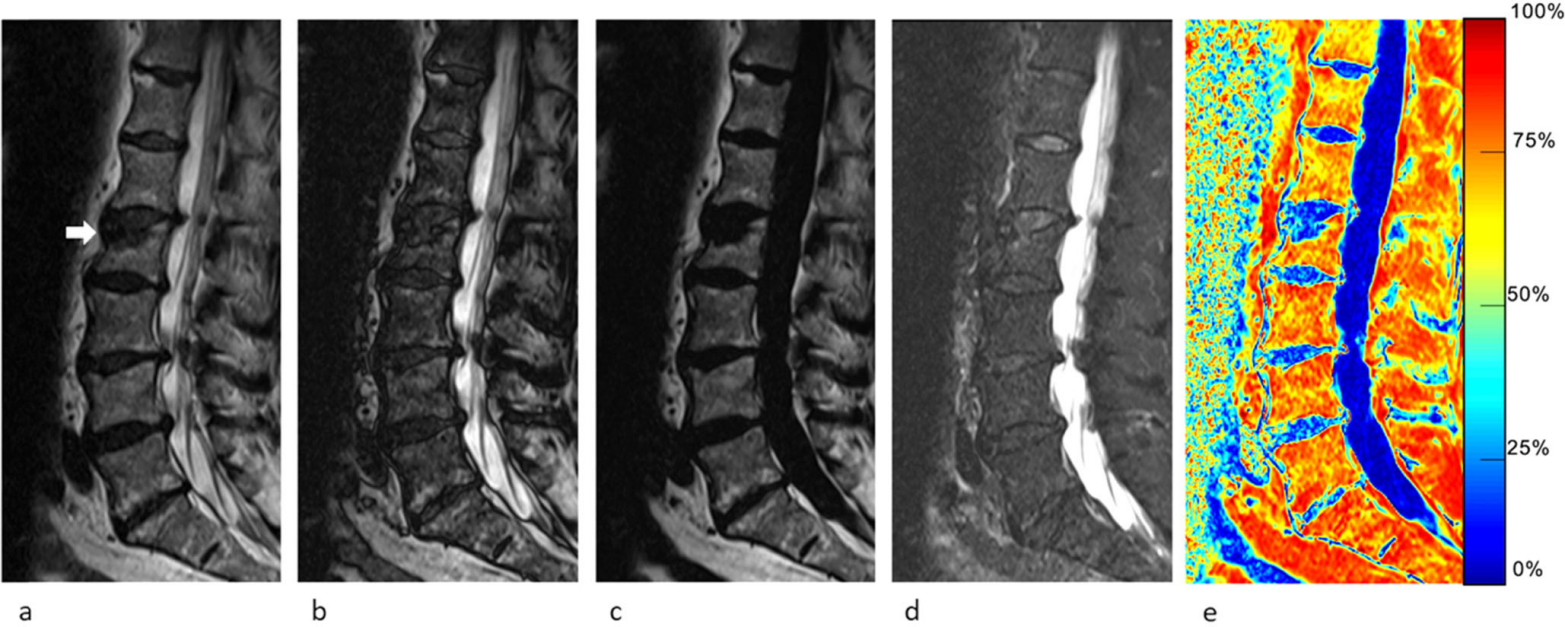
70-year-old woman with a benign vertebral compression fracture (VCF) at L2 level (arrow). Sagittal lumbar spine MR images generated from a signal FSE T2-weighted Dixon sequence include (**a**) in-phase, (**b**) out-of-phase, (**c**) fat-only, (**d**) water-only images, and (**e**) fat fraction (FF) map. At qualitative assessment, one reader wrongly qualified the VCF as malignant, but quantitative analysis correctly identified the benign nature of the VCF using the thresholds determined in this study (signal drop, FF calculated from water-only and fat-only images, and FF from maps were ROI1: 41.9%/ROI2: 40.4%; ROI1: 65.7%/ROI2: 32.3%; and ROI1: 73.9%/ROI2: 32.4%, respectively)

Qualitative image analysis was performed independently on a PACS workstations (Vue; Carestream Health) by two musculoskeletal radiologists with 2 and 11 years of experience (Y.M. and P.O.) and two neuroradiologists with 3 and 8 years of experience (S.H. and V.D.). Readers were given randomly ordered examinations and were blinded to the reference standard data (including other imaging studies and biopsy results) and to the quantitative analysis. The assessment was limited to FSE T2w Dixon image sets only (in-phase, out-of-phase, water-only, and fat-only) (Figs. 2, 3, 4, and 5). One VCF, subsequently treated by cementoplasty, was chosen per examination. Readers were asked to categorize each target VCF as benign or malignant, as they would in their clinical practice, using previously published criteria [2, 19]. No training was performed prior to the qualitative assessment.

### Quantitative assessment

Quantitative image analysis was performed by a third-year radiology resident (S.B.), under the supervision of a senior musculoskeletal radiologist with 11 years of experience (P.O.). For each target vertebra, the sagittal slice with maximum loss of height was selected. Two regions of interest (ROI) were then successively drawn freehand on the selected slice (Fig. 2). ROI1 corresponded to the entire vertebra and was drawn directly on the in-phase image and copy-pasted onto other images. ROI2 was drawn on the water-only image demarcating the area showing high signal intensity suggestive of bone marrow edema. The drawn contour was then subsequently copy-pasted onto other images. Finally, reference values for control vertebrae were obtained using a 1–2 cm diameter ROI drawn on an area of homogeneous signal intensity in a healthy vertebra closest to the VCF. ROIs were drawn on the in-phase images and copy-pasted onto the other images (Fig. 2). For each ROI, the signal intensity drops$:100\left(1-\frac{{\mathrm{Signal}}_{\mathrm{Out}-\mathrm{of}-\mathrm{Phase}}}{{\mathrm{Signal}}_{\mathrm{In}-\mathrm{Phase}}}\right)$, the FF from ROIs: $100\left(\frac{{\mathrm{Signal}}_{\mathrm{Fat}-\mathrm{Only}}}{{\mathrm{Signal}}_{\mathrm{Fat}-\mathrm{Only}}+{\mathrm{Signal}}_{\mathrm{Water}-\mathrm{Only}}}\right)$, as well as the FF from FF maps were extracted. The FF maps were generated by applying the FF equation above on a voxel-wise basis.

To assess interobserver agreement, a musculoskeletal radiologist with 2 years of experience (Y.M.) independently performed the quantitative image analysis in a subset of 30 randomly selected cases (21 benign and 9 malignant). The quantitative image analysis was performed blinded to the reference standard.

### Analysis of cases with disagreement among readers at qualitative analysis

For all cases in which at least one reader made an error at qualitative analysis in comparison to the reference standard, the quantitative assessment results were evaluated to determine if any added value in VCF categorization was present.

### Statistical analysis

Continuous variables are presented as mean ± standard deviation and categorical variables as number or percentage. Continuous variables were compared between benign and malignant VCFs using the independent samples *t*-test when the variables were normally distributed or using the Mann-Whitney test if not normally distributed. Categorical variables were compared between benign and malignant VCF using the chi-square test.

Diagnostic performance of qualitative and quantitative methods was evaluated by plotting receiver operating characteristic (ROC) curves and calculating respective sensitivity, specificity, areas under the curves (AUC), and positive and negative likelihood ratios.

For the quantitative analysis, ROC curves were plotted to determine the optimal thresholds to differentiate benign from malignant VCFs with the Liu method. Sensitivity, specificity, positive, and negative likelihood ratios were calculated using these thresholds [20]. Diagnostic performance was compared between readers and quantitative methods using pairwise non-parametric comparisons of AUCs. To assess the possible confounding effect of age and sex on quantitative measurements, we evaluated the relationship between age/sex and quantitative parameters in control vertebrae and found no significant correlation (rho = 0.02–0.09, *p* ≥ 0.4).

For the qualitative analysis, interobserver agreement was evaluated by means of Cohen’s kappa statistics and intraclass correlation coefficients (ICC) using an absolute agreement model (systematic differences between readers considered relevant) for single measures (estimating the reliability of single ratings). For the quantitative analysis, interobserver agreement was evaluated using the ICC and the Bland-Altman method. Cohen’s kappa and ICCs were interpreted as follows: ≤ 0 = poor, 0.01–0.20 = slight, 0.21–0.40 = fair, 0.41–0.60 = moderate, 0.61–0.80 = substantial, and ≥ 0.81 = almost perfect agreement.

Statistical analyses were performed using MedCalc (version 19.2.1; MedCalc Software) and Stata (Version 16; StataCorp LLC) [21]. A *p* value less than 0.05 was considered statistically significant. The Bonferroni correction was performed for multiple comparisons.

## Results

### Patient population

A total of 95 patients were included in this study and were assigned to the benign (*n* = 63) or malignant (*n* = 32) VCF groups based on the reference standard. Table 1 details the demographic data and characteristics of the VCFs.

**Table 1:** Demographic data and characteristics of vertebral compression fractures (VCF)

		Benign (*n* = 63)	Malignant (*n* = 32)	*p* value
Sex	Men	23	20	0.02^1^
	Women	40	12	
Age in years ± SD (range)		76 ± 12 (29–94)	63 ± 12 (41–84)	< 0.001^2^
Location of VCF	Cervical spine	0	2	< 0.01^1^
	Thoracic spine	21	19	
	Lumbar spine	42	11	
Histopathological confirmation	Yes	38	17	0.50^1^
	No	25	15	

^1^Chi-square test

^2^Independent samples *t*-test

### Qualitative assessment

**Fig. 6:**
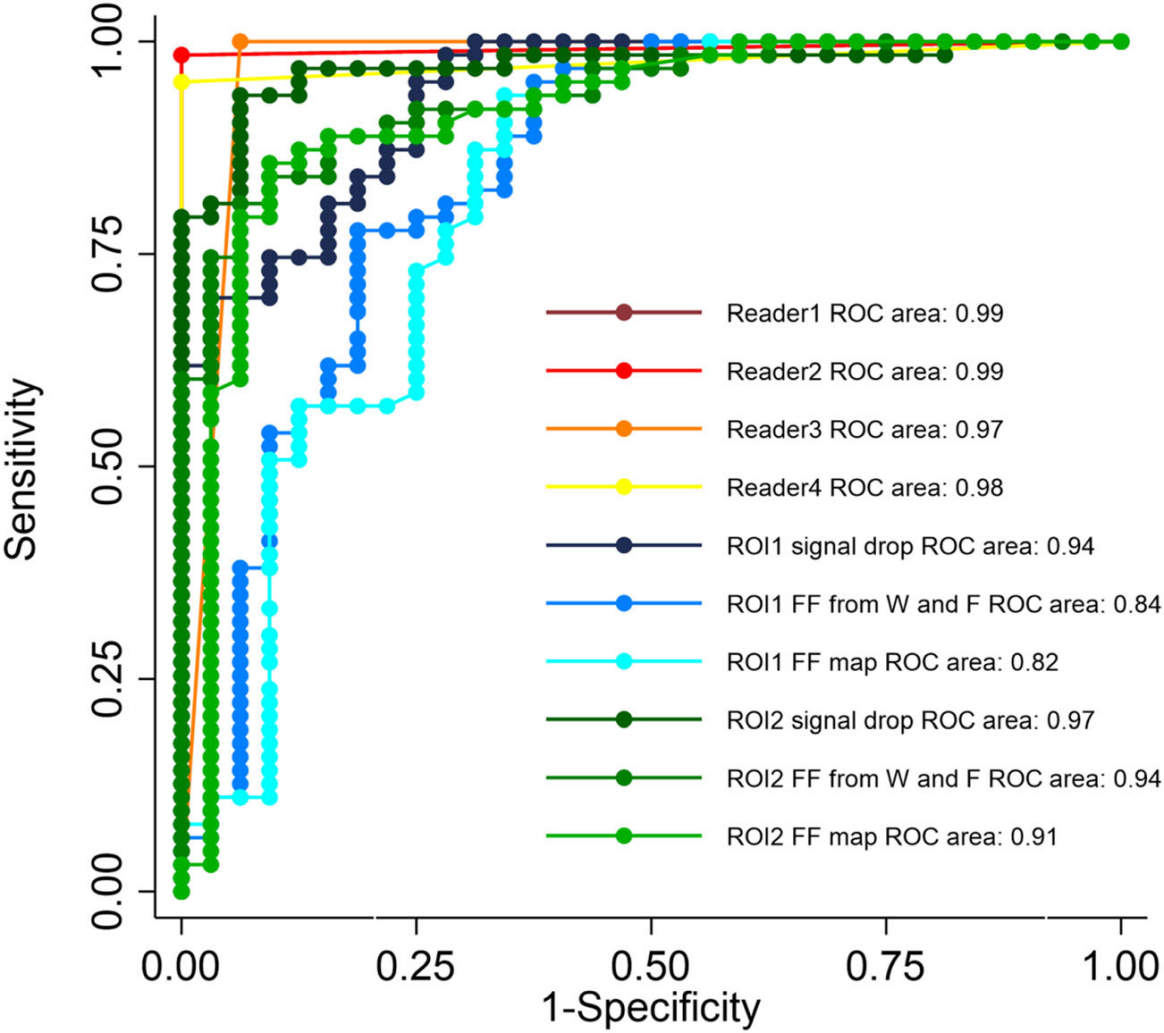
Receiver operating characteristic (ROC) curves for qualitative assessment using four readers and for six different methods of quantitative assessment of two ROIs, each with signal drop, fat fraction (FF) measured on water-only and fat-only images, as well as FF from maps, for the differentiation between benign and malignant vertebral compression fractures. Please note that the curves of readers 1 (brown) and 2 (dark red) are superimposed

Sensitivity and specificity ranged, depending on the reader, from 93.8 to 100%, and from 95.2 to 100%, respectively (Table 2). The AUCs for differentiating benign and malignant VCFs ranged from 0.97 to 0.99 (Table 2, Fig. 6). The diagnostic performance was not statistically different between readers (all *p* > 0.31, Bonferroni-corrected significance level *p* < 0.0083).

**Table 2:** Diagnostic performance of qualitative assessment of vertebral compression fractures (VCF) for each reader

	AUC [95% CI]	Sensitivity (%) [95% CI]	Specificity (%) [95% CI]	+LR	−LR
Reader 1	0.99 [0.95, 1.00]	100.0 [89.1, 100.0] (32/32)	98.4 [91.5, 100.0] (62/63)	63.0 [9.0, 440.0]	0
Reader 2	0.99 [0.95, 1.00]	100.0 [89.1, 100.0] (32/32)	98.4 [91.5, 100.0] (62/63)	63.0 [9.0, 440.0]	0
Reader 3	0.97 [0.91, 0.99]	93.8 [79.2, 99.2] (30/32)	100.0 [94.3, 100.0] (63/63)	NA	0.06 [0.0, 0.2]
Reader 4	0.98 [0.92, 1.00]	100.0 [89.1, 100.0] (32/32)	95.2 [86.7, 99.0] (60/63)	21.0 [7.0, 63.4]	0

Two-by-two differences: no statistical difference between readers (all *p* > 0.29, Bonferroni corrected significance level *p* < 0.008)

*AUC*, area under the curve; *95% CI*, 95% confidence intervals; *+LR*, positive likelihood ratio; *−LR*, negative likelihood ratio; *NA*, not applicable. Raw data are reported in parentheses wherever applicable

Interobserver agreement was almost perfect among all reader pairs and ranged from kappa = 0.88 (95% CI: 0.78, 0.98) to 1.0 (95% CI: 1.0, 1.0). The overall ICC was 0.93 (95% CI: 0.90, 0.95).

### Quantitative assessment

For all methods, the signal drop and fat fraction were higher in the benign VCF group compared to the malignant VCF group (all *p* < 0.001) (Table 3). Quantitative measures in the control vertebrae were not different between the groups (all *p* ≥ 0.55).

**Table 3:** Comparison of quantitative measurements between benign and malignant fractures

		Benign (*n* = 63)		Malignant (*n* = 32)		
Quantitative method		Mean (%)	Std dev (%)	Mean (%)	Std dev (%)	*p* value
ROI1	Signal drop	43	13	13	13	< 0.001^1^
	FF from W and F images	36	17	15	15	< 0.001^1^
	FF map	35	14	18	14	< 0.001^1^
ROI2	Signal drop	41	16	7	8	< 0.001^1^
	FF from W and F images	26	12	7	5	< 0.001^1^
	FF map	25	10	9	8	< 0.001^1^
Control vertebrae	Signal drop	49	22	52	15	0.55^1^
	FF from W and F images	77	11	76	15	0.71^1^
	FF map	77	11	77	13	0.97^1^

^1^Independent samples *t*-test

*ROI*, region of interest; *W*, water-only; *F*, fat-only

Note: ROI1 was drawn on the entire vertebra, and ROI2 was drawn on the area of the vertebra showing bone marrow edema-like signal intensity

Table 4 summarizes the diagnostic performance of the quantitative assessment including the different thresholds to discriminate malignant from benign VCFs. For ROI1, these thresholds yielded sensitivities and specificities ranging from 65.6 to 81.3%, and from 77.8 to 98.4%, respectively. For ROI2, the thresholds yielded sensitivities and specificities ranging from 90.6 to 93.8%, and from 84.1 to 93.7%, respectively. The AUCs for differentiating benign and malignant VCF ranged from 0.82 to 0.94 for ROI1 and from 0.91 to 0.97 for ROI2 (Table 4, Fig. 6). The diagnostic performance was not statistically different between quantitative methods (all *p* > 0.004, Bonferroni-corrected significance level of *p* < 0.0033).

**Table 4:** Diagnostic performance of quantitative assessment of vertebral compression fractures (VCF)

		AUC [95% CI]	Optimal cutoff for malignancy^1^	Sensitivity^2^ (%) [95% CI]	Specificity^2^ (%) [95% CI]	+LR^2^	−LR^2^
ROI1	Signal drop	0.94 [0.87, 0.98]	≤ 18%	71.9 [53.3, 86.3] (23/32)	98.4 [91.5, 100] (62/63)	45.3 [6.4,320.3]	0.3 [0.2, 0.5]
	FF from W and F images	0.84 [0.76, 0.91]	≤ 21%	81.3 [63.6, 92.8] (26/32)	77.8 [65.5, 87.3] (49/63)	3.7 [2.2, 6.0]	0.2 [0.1, 0.5]
	FF map	0.82 [0.73, 0.89]	≤ 16%	65.6 [46.8, 81.4] (21/32)	93.7 [84.5, 98.2] (59/63)	10.3 [3.9, 27.6]	0.4 [0.2, 0.6]
ROI2	Signal drop	0.97 [0.91, 0.99]	≤ 20%	93.8 [79.2, 99.2] (30/32)	93.7 [84.5, 98.2] (59/63)	14.8 [5.7,38.3]	0.1 [0.0, 0.3]
	FF from W and F images	0.94 [0.87, 0.98]	≤ 13%	90.6 [75.0, 98.0] (29/32)	84.1 [72.7, 92.1] (53/63)	5.7 [3.2, 10.2]	0.1 [0.0, 0.3]
	FF map	0.91 [0.84,0.96]	≤ 14%	90.6 [75.0, 98.0] (29/32)	85.7 [74.6, 93.3] (54/63)	6.34 [3.4, 11.7]	0.1 [0.0, 0.3]

^1^Optimal cutoff values in order to differentiate benign from malignant VCFs corresponding with the Liu method

^2^Sensitivity, specificity, +LR, and −LR calculated for optimal cutoff for malignancy

*AUC*, area under the curve; *95% CI*, 95% confidence interval; *+LR*, positive likelihood ratio; *−LR*, negative likelihood ratio; *FF*, fat fraction; *ROI*, region of interest. Raw data are reported in parentheses wherever applicable

Interobserver agreement for the VCF quantitative analysis was excellent for all methods (ICC ranging from 0.89 [95% CI: 0.78, 0.95] to 0.96 [95% CI: 0.92, 0.98]). No systematic bias was found (mean difference ranging from − 1.3 [95% CI: − 2.9, 0.3] to − 0.0 [95% CI: − 2.9, 1.9], all *p* ≥ 0.10).

### Comparison of qualitative and quantitative assessment

Pairwise comparisons did not show any statistical difference in diagnostic performance between qualitative and quantitative methods (all *p* > 0.0013, Bonferroni corrected significance level of *p* < 0.0011).

### Analysis of cases with disagreement among readers at qualitative analysis

There were five cases in which at least one reader made an error at qualitative analysis. Out of these five cases, the quantitative assessment using the aforementioned thresholds (Table 4) correctly categorized the lesion in all five cases using ROI2, regardless of the quantitative method used (Fig. 5).

## Discussion

In this study, we have shown that a single FSE T2w Dixon sequence provided high diagnostic performance and high interobserver agreement for the differentiation between benign and malignant VCFs, both at qualitative analysis with multiple readers, and quantitative analysis with various measurement methods.

The diagnostic performance for the differentiation of benign and malignant VCFs through qualitative analysis of a single FSE T2w Dixon sequence was comparable to that previously reported with other sequences. In particular, a recent meta-analysis of eighteen studies on this topic found a pooled sensitivity, specificity, and AUC of 89% (95% CI: 86, 92%), 88% (95% CI: 85, 91%), and 0.95, respectively [4]. In light of these results, a single FSE T2w Dixon sequence could be used instead of the set of sequences which are usually acquired in the sagittal plane for this indication, as has been previously shown for spine MRI protocols for the detection of metastases or for the workup of non-specific low back pain or lumbar radiculopathy [11, 12]. Using a single sagittal FSE T2w Dixon sequence, the morphological information is provided by the in-phase images, while the fat-only images and water-only images may replace T1-weighted and fat-suppressed fluid-sensitive sequences for the analysis of fat and fluid signal, respectively.

Furthermore, we showed that the same FSE T2w Dixon sequence provided high diagnostic performance in the differentiation of benign and malignant VCFs through quantitative analysis. While very few reports of quantitative analysis of spin echo–based Dixon sequences exist for the characterization of bone marrow lesions, quantitative chemical shift imaging has been previously validated with gradient echo–based sequences, including for the characterization of VCFs [22, 23]. In a meta-analysis, Thawait et al found one study showing a sensitivity and specificity of 95% (95% CI: 81, 99) and 89% (95% CI: 81, 93), respectively, with a malignancy threshold of signal drop < 20% [2]. More recently, Kim et al and Schmeel et al evaluated a six-echo 3D gradient echo–modified Dixon sequence and reached an AUC of 0.98 in both studies for the differentiation of benign and malignant fractures, using a threshold of FF < 5.3% and ≤ 9% for malignancy, respectively [14, 24]. The use of these gradient echo sequences however requires their acquisition in addition to spin echo–based sequences that form the basis for the morphological assessment of bone marrow with MRI [22, 25, 26], whereas a FSE Dixon sequence can provide both the morphological information and reliable quantitative assessment of the fat fraction.

The diagnostic performance of the quantitative analysis varied depending on the measurement method. Signal drop measurement with ROI2, with a threshold for malignancy of ≤ 20%, provided the best diagnostic performance (AUC = 0.97). Additionally, ROI2 is relatively easy and fast to draw, with excellent interobserver agreement. Finally, there is no need to generate additional fat fraction maps with this method, since the analysis is performed on native in-phase and out-of-phase images.

Limitations of the quantitative analysis of Dixon sequences have been reported and mainly include lack of specificity in conditions leading to underestimated fat content (i.e., hyperostosis due to healing fractures) [25, 27]. Retrospectively, in our cohort, 6/6 false positives of signal drop with ROI2 were subacute VCFs present for at least two weeks prior to MRI, some associated with hyperostosis or clefts visible at CT. All of these were correctly diagnosed as benign by all readers. The poorer specificity of quantitative Dixon imaging in case of subacute VCFs should be kept in mind to avoid overdiagnosis.

To our knowledge, qualitative and quantitative assessments for the characterization of VCFs have never been compared. In clinical practice, qualitative analysis is fast and allows accurate differentiation between benign and malignant VCFs in the majority of cases. However, in uncertain cases, quantitative information can be readily post-processed from the set of images provided by the FSE T2w Dixon sequence, and be used as an additional diagnostic criterion for the differentiation between benign and malignant VCFs, complementary to morphological criteria.

The strengths of our study, compared to previous reports on the characterization of VCFs, include strict criteria to categorize VCFs as benign or malignant. In particular, more than half of cases were histologically proven and, in order to avoid false negatives, we excluded all patients with a history of malignancy, and followed up benign cases for nine months or more. Second, we provide a precise description of the different measurement methods, which was not always the case in previous studies [16]. Third, we provide a comparison of the different quantitative methods previously reported in isolation in the literature (signal drop, FF from water and fat images, FF maps) [14–17, 22, 24, 25, 27]. Finally, our sample size of 95 cases was larger than the largest cohort previously reported (57 patients, including 25 with malignant VCF) and we analyzed only one VCF per patient to avoid clustered data [14].

The limitations of our study include a potential bias related to the fact that we only included patients treated for cementoplasty at our institution, a center specializing in oncological treatment, which artificially increased the percentage of malignant VCFs compared to the general population. Second, we did not correct for the potential confounding effect of previous treatment, either chemotherapy or radiotherapy, which may alter the signal and fat fraction of bone marrow. Third, the retrospective design intrinsically leads to limitations, which we tried to minimize through strict criteria for patient selection and for the reference standard. Finally, these results should be confirmed in a multicentric study involving different manufacturers. Indeed, many versions of the Dixon sequence exist, and all may not perform identically.

In conclusion, a single FSE T2w Dixon sequence can be used to differentiate benign and malignant vertebral compression fractures with high diagnostic performance and high interobserver agreement and provides both qualitative and quantitative information that can be complementary in the evaluation. The use of this simplified protocol may reduce acquisition time and improve patient comfort.

## Abbreviations

95% CI: 95% confidence interval
AUC: Area under the receiver operating characteristic curve
CHESS: Chemical shift selective
FF: Fat fraction
FSE: Fast spin echo
ICC: Intraclass correlation coefficient
MRI: Magnetic resonance imaging
ROC: Receiver operating characteristic
ROI: Region of interest
STIR: Short-tau inversion recovery
VCF: Vertebral compression fracture

## Declarations

### Guarantor

The scientific guarantor of this publication is Dr Patrick Omoumi.

### Statistics and biometry

Two of the authors has significant statistical expertise.

### Methodology

• diagnostic or prognostic study

• performed at one institution
